# High Anti–Phenolic Glycolipid-I IgM Titers and Hidden Leprosy Cases, Amazon Region

**DOI:** 10.3201/eid1805.111018

**Published:** 2012-05

**Authors:** Claudio Guedes Salgado, Denis Vieira Gomes Ferreira, Marco Andrey Cipriani Frade, Layana de Souza Guimarães, Moisés Batista da Silva, Josafá Gonçalves Barreto

**Affiliations:** Federal University of Pará, Marituba, Brazil (C.G. Salgado, D.V.G. Ferreira, L.S. Guimarães, M.B. Silva, J.G. Barreto);; Federal University of Pará, Belém, Brazil (C.G. Salgado, M.B. Silva);; São Paulo University–Faculty of Medicine, São Paulo, Brazil (M.A.C. Frade);; Dr Marcello Candia Reference Unit in Sanitary Dermatology of the State of Pará, Marituba (L.S. Guimarães);; Federal University of Pará, Castanhal, Brazil (J.G. Barreto)

**Keywords:** leprosy, serology, PGL-I, subclinical infection, bacteria, Hansen disease, Mycobacterium leprae, Amazon Region, IgM, titers, seroprevalence

**To the Editor:** Leprosy remains a serious public health issue. Although the World Health Organization elimination target was achieved in 2000, with a prevalence of <1 case/10,000 persons, despite progress since introduction of multidrug therapy (*1*), large pockets of poverty remain in which the disease is hyperendemic and underdiagnosed. In fact, in highly disease-endemic areas, the prevalence of previously undiagnosed leprosy cases in the general population has been reported to be 6× higher than the registered prevalence (*2*).

Most leprosy patients are in India and Brazil. In Brazil, new cases are concentrated in the Northeast, Midwest, and Amazon regions (from state capitals to the inner counties). Access to the health system is poor in these regions because of severe inequalities in the public health system of Brazil (*3*),

A total of 34,894 new cases were registered in Brazil during 2010 (*4*), corresponding to an incidence rate of 18.22 cases per 100,000 population. Pará State accounted for 10.2% of cases (3,562 cases), an incidence rate of 46.93 per 100,000 population. When only children <15 years of age were considered, Pará registered 389 new cases of leprosy in 2010, representing 10.9% of all cases, an incidence rate of 16.52 per 100,000 population. In Oriximiná, a county with 62,794 inhabitants in northwestern Pará, ≈800 km from Belém, Pará’s capital, a mean of 13.8 cases per year were registered for the past 5 years.

In 2010, in Oriximiná, we collected plasma samples from 138 students 8–18 years of age, from 35 leprosy patients who received a diagnosis during 2004–2009, and from 126 contacts of these patients (Federal University of Pará Research Ethics Committee protocol no. 197/07). We tested all of these samples for anti–phenolic glycolipid-I (PGL-I) IgM; 42% of students, 54.3% of case-patients, and 45% of case-patient contacts were seropositive. In addition to collecting samples, we clinically examined the leprosy patients and their contacts, among whom we identified 3 new leprosy cases. We did not examine students at that time. Contacts were persons from the same household or neighborhood whom the index case-patient described as a person with whom he or she had a close relationship. Leprosy cases were diagnosed in the field on the basis of clinical signs, loss of sensation on the skin lesions, and presence of enlarged nerves. For operational reasons, skin smears were not performed. All cases were diagnosed by 2 leprologists. We used the Ridley-Jopling classification, associated with the indeterminate clinical type, as defined by the Madrid classification. The ELISA cutoff for positive results was arbitrarily established as an optical density of 0.295 based on the average plus 3× the SD of the test results from 14 healthy persons from the Amazon region (*5*).

Because studies of the seroprevalence among contacts have reported a proportion of seropositive persons ranging from ≈1.9% to 18.4% (*6*), we returned to Oriximiná 16 months after the first visit. We examined 2 groups of students and their contacts; 1 group was positive for anti–PGL-I, and the other group was negative for anti–PGL-I. We visited 44 households in 1 week. From the 35 leprosy patients encountered during the first visit, we selected 25 households to survey (14 with an anti–PGL-I–positive contact in the household and 11 without), and among students with results of anti–PGL-I serology, we selected 19 households (11 positive with an anti–PGL-I–positive contact in the household and 8 without). During our visits to all of these households, we examined 222 persons (Table).

**Table:** New leprosy cases detected among selected households, Oriximiná, Pará State, Brazilian Amazon, 2010

Group	Household contact anti–PGL-I IgM ELISA result	No. households visited	No. persons examined	No. new cases		
				Among persons previously tested	Among contacts of persons previously tested	Total†
Leprosy patients	Positive	14	43	9	4	13
	Negative	11	42	1	1	2
Students	Positive	11	84	5	5	10
	Negative	8	53	1	4	5
Total		44	222	16	14	30

*Households were selected from among 35 leprosy patients encountered during the first visit (25 households, 14 with an anti–PGL-I–positive contact in the household and 11 without) and among students with results of anti–PGL-I serology (19 households, 11 with an anti–PGL-I–positive contact in the household and 8 without). PGL-I, phenolic glycolipid-I. †Fisher exact test comparing case-patients and non–case-patients among those positive or negative for anti-PGL-I IgM revealed a statistically significant difference (p = 0.0280).

When we arrived in Oriximiná, only 2 cases had been registered in the national notifiable diseases information system. By using our approach, 23 new cases were found after we investigated households that had a person positive for anti–PGL-I (15 multibacillary, 8 paucibacillary); we found only 7 new cases in households where residents were negative for anti–PGL-I (4 multibacillary, 3 paucibacillary) (Table). For comparison, during the last traditional leprosy campaign in Oriximiná in 2008, eight new cases were detected. Furthermore, by using our strategy, the local public health service detected 9 additional new cases during the 4 months after our departure from Oriximiná.

These data emphasize that contact examination is crucial for identifying new cases (*7*) and that such investigation must be conducted periodically. Our data also indicate that subclinical infections are highly prevalent among public school students in the Amazon region and that identifying students with positive anti–PGL-I test results can lead to discovery of new leprosy cases among students’ household contacts.
